# Magnetically recoverable magnetite-reduced graphene oxide as a demulsifier for surfactant stabilized crude oil-in-water emulsion

**DOI:** 10.1371/journal.pone.0232490

**Published:** 2020-04-30

**Authors:** Xin Hui Yau, Cheng Seong Khe, Mohamed Shuaib Mohamed Saheed, Chin Wei Lai, Kok Yeow You, Wai Kian Tan

**Affiliations:** 1 Department of Fundamental and Applied Sciences, Universiti Teknologi PETRONAS, Seri Iskandar, Perak, Malaysia; 2 Centre of Innovative Nanostructures and Nanodevices (COINN), Universiti Teknologi PETRONAS, Seri Iskandar,Perak, Malaysia; 3 Nanotechnology & Catalysis Research Centre (NANOCAT), Institute for Advanced Studies (IAS), University of Malaya, Kuala Lumpur, Malaysia; 4 Department of Communication Engineering, Faculty of Electrical Engineering, Universiti Teknologi Malaysia, Skudai, Johor, Malaysia; 5 Institute of Liberal Arts and Sciences, Toyohashi University of Technology, Toyohashi, Aichi, Japan; University of Salento, ITALY

## Abstract

Oily wastewater, especially water-oil emulsion has become serious environmental issue and received global attention. Chemical demulsifiers are widely used to treat oil-water emulsion, but the toxicity, non-recyclable and non-environmental friendly characteristic of chemical demulsifiers had limited their practical application in oil-water separation. Therefore, it is imperative to develop an efficient, simple, eco-friendly and recyclable demulsifiers for breaking up the emulsions from the oily wastewater. In this study, a magnetic demulsifier, magnetite-reduced graphene oxide (M-rGO) nanocomposites were proposed as a recyclable demulsifier to break up the surfactant stabilized crude oil-in-water (O/W) emulsion. M-rGO nanocomposites were prepared via in situ chemical synthesis by using only one type Fe salt and GO solid as precursor at room temperature. The prepared composites were fully characterized by various techniques. The effect of demulsifier dosage and pH of emulsion on demulsification efficiency (*E*_D_) has been studied in detailed. The demulsification mechanism was also proposed in this study. Results showed that M-rGO nanocomposites were able to demulsify crude O/W emulsion. The *E*_D_ reaches 99.48% when 0.050 wt.% of M-rGO nanocomposites were added to crude O/W emulsion (pH = 4). Besides, M-rGO nanocomposites can be recycled up to 7 cycles without showing a significant change in terms of *E*_D_. Thus, M-rGO nanocomposite is a promising demulsifier for surfactant stabilized crude O/W emulsion.

## Introduction

Nowadays, oil pollution has become a serious environmental issue and received global attention. Oily wastewaters are usually made up of free oil, soluble oil and water-oil emulsion [1]. Free oil (oil droplet size > 150 μm) refers to a considerable amount of oil that is present as a separated phase. Soluble oil (oil droplet size within 20–150 μm) refers to small amount of oil dispersed in water, whereas oil-water emulsion (oil droplet size < 20 μm) is formed when one phase (either oil phase or water phase) is dispersed in the other phase [2]. Oil-water emulsion is harder to treat as compared to free oil and soluble oil, especially when the emulsions are stabilized by particles or surfactants. The presence of hazardous components in emulsion can cause both environment pollution [3] and threaten human health by entering into food chain [4]. Conventional techniques, such as gravity separation, flotation, coagulation, ultra-centrifugation and membrane filtration have been used to separate oil from oil-in water (O/W) emulsions [5, 6]. However, these methods are inefficiently in separation of oil from O/W emulsion, complicated instrument setup and high energy consumption.

Chemical demulsification is the most common approach for treating emulsified oil-water mixture as the chemical additives can speed up the emulsion breaking process [7]. Up to date, chemical demulsifiers, such as alkoxylated amines, alkoxylated epoxy resins, alkyl-phenol formaldehyde resins, copolymers of polypropylene oxide-polyethylene oxide [8], poly(maleic anhydride-alt-1-dodecene) with polyethylene glycol (PEG 1000) and polypropylene glycol (PPG1000) [9], polyamidoamine-polyether [10], ethylcellulose polymer [11], 2-amino-5-dedocyl benzene sulfonic acid surfactant [12], etc. have been used for breaking oil-water emulsion in oil industry. However, these chemical demulsifiers may generate secondary pollutants, toxic and cannot be recycled after demulsification, limiting their practical application in oil-water separation. Therefore, it is imperative to develop an efficient, eco-friendly and recyclable demulsifiers for treating the emulsions from the oily wastewater.

In recent years, magnetic materials have emerged as a new generation of demulsifier due to their magnetic response to external magnetic field and easy separation from a complex multiphase system by an external magnetic field [13]. The magnetic based demulsifier is more selective than chemical demulsifier as it can speed up the demulsification rate with the aid of applied magnetic field [14]. Magnetic based demulsifiers are more environmental friendly and cost effective than chemical demulsifiers as it can be recycle through magnetic separation and reuse, and no generation of secondary pollution [15]. Among various magnetic nanoparticles used, magnetite (Fe_3_O_4_) is considered to be an ideal candidate because of its low toxicity to the human health and environment friendly in nature [16]. However, key drawback of magnetite nanoparticles is heavy aggregation which may consequently affect their magnetic properties and structural stability [17].

Graphene, a two-dimensional crystalline material, consists of one atom thick planar sheet of sp^2^ bonded carbon structure in a honeycomb crystal lattice [18]. Graphene has peculiar properties such as high specific surface area (theoretically 2630 m^2^g^-1^ for monolayer graphene), excellent thermal conductivity (~5000 W mK^-1^), high electron mobility (200000 cm^2^ vs^-1^), high optical transmittance (~97.7%), and optimum mechanical properties (~1.0 TPa) [18–20]. Graphene oxide (GO) is the precursor for graphene synthesis by either chemical or thermal reduction processes [21]. GO is synthesized through oxidation of graphite using oxidants. GO consists various oxygen-containing functional groups, including epoxy, hydroxyl, carbonyl and carboxyl groups. Hence, GO is highly hydrophilic and stably dispersed in water [22]. In contrast, the reduced graphene oxide (rGO) is hydrophobic due to the removal of oxygen atoms, thus it tends to aggregate or be irreversibly restacked [23]. Graphene, GO and rGO have been commonly used as a template for the synthesis of a variety of metal and metal oxide nanoparticles due to its extraordinary properties which result in the formation of composite materials, where they (graphene, GO and rGO) have no chances of leaching in the aqueous and solvent medium [16]. By constructing a magnetite reduced graphene oxide (M-rGO) nanocomposites, heavy aggregation of both magnetite nanoparticles and rGO nanocomposites can be reduced. Besides, the M-rGO nanocomposites displayed a large surface area, good dispersion, magnetic responsive, easy separation and potential recyclability. Liang et al. [24] fabricated Fe_3_O_4_/rGO nanocomposites through co-precipitation to develop high-performance lithium-ion batteries. Hoan et al. [25] synthesized Fe_3_O_4_/rGO nanocomposites using ethanol as solvent in solvothermal method under nitrogen atmosphere, and used to remove toxic heavy metals from aqueous solution. Sharif et al. [26] synthesized rGO/Fe_3_O_4_ composite using hydrazine-hydrothermal method, and used to remove methylene blue from water. Thus far, M-rGO nanocomposites have been widely used for electrochemical application and the removal of dyes and heavy metals from wastewater, but the study of M-rGO nanocomposites as demulsifier in oil-water separation is scarce. In 2016, Ma et al. [27] had synthesized ODTS modified M-rGO nanocomposites (MRGO@ODTS) to demulsify both water-in-oil (W/O) and oil-in-water (O/W) emulsions. Ma et al. [27] achieved demulsification efficiency of 83.8 ± 4.1% and 52.0 ± 2.8% when 0.05 g M-rGO@ODTS were added to both W/O and O/W emulsions, respectively. In this study, M-rGO nanocomposites are synthesized without any surface modification and used to treat highly stable crude O/W emulsion. It is believed that M-rGO without any surface modification has great demulsification performance.

The current study aims to elaborate on the following issues:

- The one step synthesis of magnetite-reduced graphene oxide (M-rGO) nanocomposites using only one type Fe salt and GO solid as precursor at room temperature.
- The capability of M-rGO nanocomposites as demulsifier for surfactant stabilized crude O/W emulsion.
- The plausible mechanism of demulsification of surfactant stabilized crude oil-in-water emulsion.
- The reusability of M-rGO nanocomposites.

The ultimate goal of the current study is to prepare a simple, effective, eco-friendly and recyclable demulsifier which can break down the surfactant stabilized crude oil-in-water emulsion in short time, at room temperature and which can be reuse for multiple times.

## Experimental section

### Materials

Graphite powder (<20 μm, ≥99.99%) was purchased from Sigma Aldrich, USA. Sulphuric acid (H_2_SO_4_, 95–97%), phosphoric acid (H_3_PO_4_, 85%), potassium permanganate (KMnO_4_, 99.9%), hydrogen peroxide (H_2_O_2_, 30%), hydrochloric acid (HCl, 37%), ammonium hydroxide (NH_4_OH, 25%), iron (II) sulphate heptahydrate (FeSO_4_·7H_2_O, 99.5%), Tween 60 and sodium hydroxide (NaOH, ≥97.0%) were supplied by Merck, Germany. All of the chemical used for experiments were of analytical grade, except Tween 60 (chemically pure), and were used as received without further purification. Tapis crude oil was obtained from the Petronas Refinery at Melaka, Malaysia. The deionized water was obtained using a Millipore Milli-Q water purification system with a resistivity of 18.2 MΩ cm^-1^ and used throughout the experiment.

### Preparation of graphene oxide (GO)

GO was synthesized from graphite powder by using improved Hummers method [28]. In a typical synthesis, 3 g of graphite powder was added to a mixture of 360 mL of H_2_SO_4_, 40 mL H_3_PO_4_ and 18.0 g of KMnO_4_, following by 1 h stirring in an ice bath condition. The ice bath was discarded, and a water bath was prepared. The mixture was stirred at 50°C for 72 hours. Then 400 mL ice cubes (made from deionized water) and 8 mL of H_2_O_2_ were added to the mixtures until its colour was changed to bright yellow. The mixture was washed with 1M HCl for 24 hours. The mixture was again centrifuged and washed with deionized water. Finally, the samples GO were obtained and dried at 40°C.

### Preparation of magnetite reduced graphene oxide (M-rGO) nanocomposites

M-rGO nanocomposites were synthesized by in situ chemical synthesis [17]. A mass of 0.2 g of GO was dispersed in 200 mL of deionized water. NH_4_OH was then added to the GO solution until pH 11–12 was obtained. Meanwhile, 4.0g FeSO_4_·7H_2_O was dissolved in 100 mL deionized water. Subsequently, the GO solution was mixed with as-prepared solution of FeSO_4_, and stirred at different durations (3, 6, 12, 18, 24 h) at room temperature. Black solution with precipitates was obtained. The suspension was centrifuged and washed thrice with deionized water, and then dried at 40°C. The final products were M-rGO nanocomposites. The prepared M-rGO nanocomposites with different stirring durations and their designations are listed in Table 1.

**Table 1:** Synthesis parameter and designations of M-rGO.

Sample	Stirring Duration (hours)
M-rGO3	3
M-rGO6	6
M-rGO12	12
M-rGO18	18
M-rGO24	24

### Preparation of crude oil-in-water (O/W) emulsion

The mother crude O/W emulsion was prepared by mixing 1 mL Tapis crude oil, 2.5 g Tween 60, and 76 mL deionized water with a high-speed mixer for 10 minutes until the oil and water phases were completely homogeneous. The O/W emulsion used for the demulsification test was prepared by diluting 2 wt.% mother crude O/W emulsion with 98 wt.% deionized water. The O/W emulsion was then transferred into a bottle and left for a week at room temperature to check its stability.

### Demulsification test

Different amounts (0.005–0.050 wt.%) of M-rGO nanocomposites were added to 20 ml O/W emulsions and mechanically mixed using a Stuart SF1 flask shaker at 400 oscillations min^-1^ for 10 mins at 25°C. The mixtures were allowed to settle for 2 mins at room temperature, and M-rGO nanocomposites were then removed by a 3000 Gs NdFeB magnet. The concentration of oil in separated water after demulsification test was measured at 257 nm using a spectrophotometric technique and compared with a standard curve obtained through a series of standard emulsion with different oil concentrations. The demulsification efficiency is calculated as Eq 1:

$$E_{D}=\left[\left(c_{o}‑c_{i}\right)/c_{o}\right]x100\%$$ (1)

where *E*_D_ is the demulsification efficiency (%), *c*_o_ is the initial oil content (mg L^−1^), and *c*_i_ is the residual oil content (mg L^−1^) in the separated water.

The effect of pH on the demulsification efficiency was investigated by varying the pH value of the diluted O/W emulsion using 0.5 M HCl and 0.5 M NaOH. All experiments were conducted in triplicate, and the reported *E*_D_ values were the average of three repetitions. Blank test also performed for the O/W emulsions without addition of M-rGO nanocomposites. The standard deviation of the demulsification test was less than 4%.

### Recycle test

After demulsification test, M-rGO nanocomposites were collected using 3000 Gs NdFeB magnet, and then repeated washed with ethanol and deionized water for 6 times to remove the attached oil. Regenerated M-rGO nanocomposites were then dried at 40°C for 1 h and reused for the next demulsification test. Seven cycles were performed to assess the reusability of M-rGO nanocomposites.

### Material characterization

The morphology of as-prepared samples was characterized by field emission scanning electron microscopy (FESEM), using ZEISS Leo Supra 55 variable pressure FESEM with an accelerating voltage of 5 kV. FESEM-EDX (energy-dispersive X-ray, EDAX ZEISS Leo Supra 55) mapping was used to investigate the presence and attachment magnetite nanoparticles on the surface of rGO sheets. X-ray diffraction (XRD) measurements were carried out using Bruker D8 advance diffractometer with Cu Kα radiation (λ = 1.5406 Å) in the range of 2θ = 5^o^-80^o^. Raman spectra were recorded using a Horiba Jobin Yvon HR800 Raman spectroscope equipped with a 514.5 nm laser. Fourier transform infrared (FTIR) spectra were recorded with Perkin Elmer Spectrum BX FTIR with a wavenumber range of 400–4000 cm^-1^. X-ray photoelectron spectroscopy (XPS) measurements were carried out on a Thermo Scientific K-Alpha instrument with Al Kα (*hv* = 1468.6 eV) as the radiation source. Vibrating sample magnetometer (VSM) (DMS Model 10) was utilized to investigate the magnetic properties of M-rGO nanocomposites. The concentration of the oil after demulsification was determined using Cary 100 UV-Vis spectrophotometer (dual-beam mode). Optical microscopy image of O/W emulsion were taken by a polarizing microscope (Leica, DM1000) equipped with a digital camera (Dino-Eye Edge). The surface charge of M-rGO nanocomposites and O/W emulsions and the droplet size of crude O/W emulsion were determined using Malvern Instruments Zetasizer Nano ZS with 633 nm laser.

## Results and discussion

### Characterization of M-rGO nanocomposites

**Fig 1:**
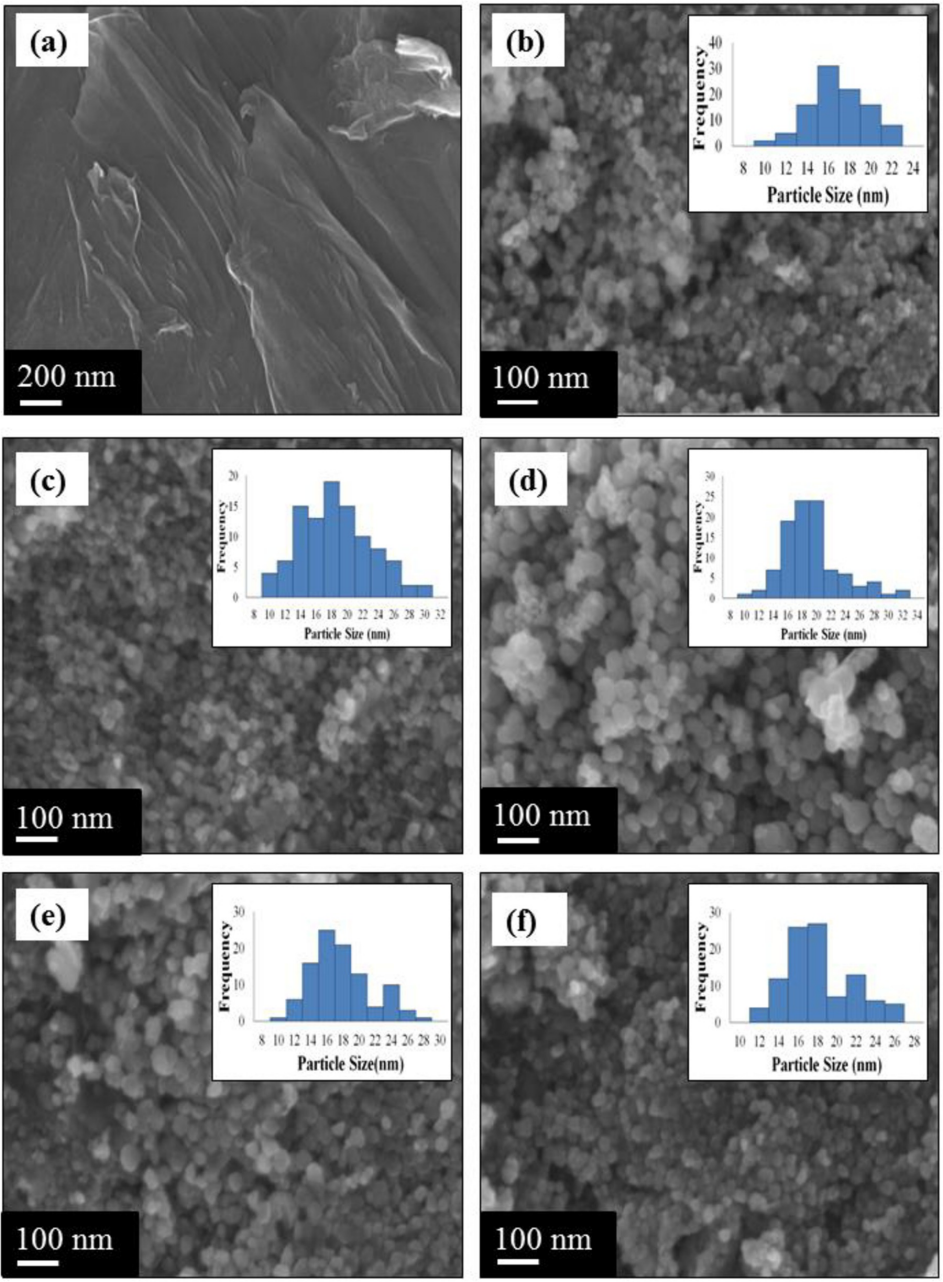
FESEM image of (a) GO. FESEM images and particle size histogram (inset) of (b) M-rGO3, (c) M-rGO6, (d) M-rGO12, (e) M-rGO18 and (f) M-rGO24.

FESEM was employed to study the surface morphology and particle size of the M-rGO nanocomposites. Fig 1(A) depicts the representative FESEM image of GO. It was found that GO sheets revealed a rough and irregular wrinkled structure, which might have resulted from the introduction of oxygen-containing functional groups, including epoxy, hydroxyl, carbonyl, and carboxyl groups, during the oxidation of graphite by the improved Hummers method. (Fig 1B–1F) present the FESEM images and particle size histograms of the M-rGO nanocomposites synthesized with various stirring durations. In general, the FESEM images of M-rGO (regardless of the stirring duration) in (Fig 1B–1F) displayed plenty of nanoparticles spread on the surface of rGO sheets. These magnetite nanoparticles were tightly joined together on the surface of rGO sheets, forming a bumpy and coarsen layer. Therefore, the M-rGO nanocomposites were successfully synthesized regardless of the stirring duration. Besides, as shown in (Fig 1B–1F), the amounts of magnetite nanoparticles loaded on the surface of rGO sheets did not significantly change with prolonged stirring duration from 3 h to 24 h. The possible reason is that the anchor site of rGO sheets was almost fully occupied by magnetite nanoparticles even at 3 h of stirring duration, as shown in Fig 1(B). In addition, as the stirring duration was increased, the magnetite nanoparticles on the surface of rGO sheets became prone to conjoin with neighboring particles and agglomerate, resulting in a larger particle size [29]. The reason is that small-sized magnetite nanoparticles that exhibit a large surface-area-to-volume ratio and high surface energies tended to agglomerate easily [30]. Although rGO sheets prevented the agglomeration of magnetite nanoparticles, slight agglomeration still occurred. The rGO sheets were covalently bonded with magnetite nanoparticles, thereby reducing the likelihood of agglomeration and resulting in a more uniform particle size of magnetite nanoparticles. The agglomeration and uniform particle size of magnetite nanoparticles were further confirmed by the size distribution histogram.

In this study, the average particle size of magnetite nanoparticles was measured and presented in a frequency histogram. A total of 100 particles were selected from the FESEM images and used to analyze the particle size distribution. The average particle sizes of magnetite nanoparticles for M-rGO3, M-rGO6, M-rGO12, M-rGO18, and M-rGO24 were approximately 15.91±2.74, 17.46±4.50, 18.29±4.18, 22.34±6.28, and 17.16±3.40 nm, respectively. M-rGO3 had the smallest particle size and standard deviation. A smaller value of standard deviation corresponded to a narrower particle size distribution and therefore a more uniform size of magnetite nanoparticles. Thus, combining the analysis of the FESEM images and the particles size distribution histogram suggests that M-rGO3 or M-rGO synthesized with 3 h of stirring duration had adequate, smaller, and uniformly sized magnetite nanoparticles loaded on the surface of rGO sheets.

Table 2 shows the EDX analysis (in At.%) of the GO and M-rGO samples in terms of C, O, Fe, carbon-to-oxygen composition C/O ratio, and iron-to-carbon composition (Fe/C) ratio. In general, GO consists only of C and O elements, whereas M-rGO nanocomposites (regardless of the stirring duration) consist of C, O, and Fe elements. The existence of Fe element in the M-rGO samples implied the successful attachment of magnetite nanoparticles on the rGO surface. This result will be combined with the XRD results in a subsequent section to prove the presence of magnetite nanoparticles on the surface of rGO sheets. The element distributions of C, O, and Fe in the M-rGO nanocomposites were in the range of 21–23, 52–58, and 20–26 At.%, respectively. The stirring duration imposed no significant effect on the element distribution. The carbon content (21–23 At.%) of M-rGO nanocomposites in the present study was close to that found by Li et al. [31]. The high atomic percentage of the O element may be due to the presence of oxygen atoms in both rGO and magnetite nanoparticles. The C/O ratio of GO was 1.17, indicating that GO contained a high density of oxygen moieties (46.07 At.%) on its surface [32]. By contrast, the C/O ratio of M-rGO samples was not measured as it was inapplicable in this case (the O element may originate from rGO, magnetite, or both). Thus, the Fe/C ratio was introduced to ascertain the degree of loading magnetite nanoparticles on the surface of rGO sheets. The EDX results revealed that M-rGO3 had the highest Fe/C ratio (1.20). This result confirmed that 3 h of stirring duration was sufficient for the attachment of magnetite nanoparticles (25.75 At.%) on the surface of rGO sheets (21.46 At.%).

**Table 2:** EDX analysis (in At.%) of GO and M-rGO nanocomposites.

Sample	C (At.%)	O (At.%)	Fe (At.%)	C/O ratio	Fe/C ratio
GO	53.93	46.07	N/A	1.17	N/A
M-rGO3	21.46	52.79	25.75	N/A	1.20
M-rGO6	21.88	57.73	20.39	N/A	0.93
Mr-GO12	22.80	53.58	23.62	N/A	1.04
Mr-GO18	21.91	54.31	23.78	N/A	1.09
Mr-GO24	23.30	51.93	24.77	N/A	1.06

**Fig 2:**
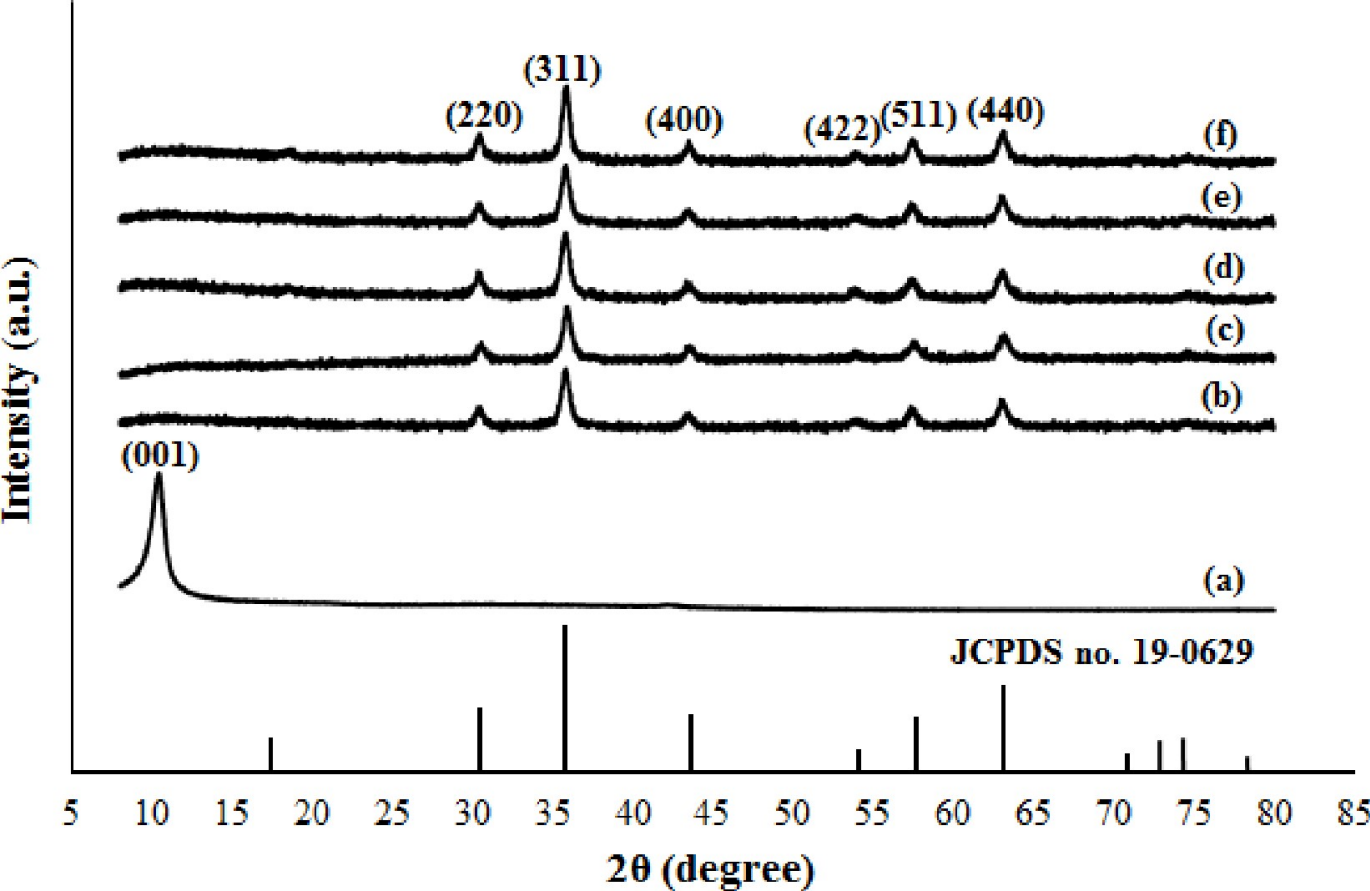
XRD patterns of (a) GO, (b) M-rGO3, (c) M-rGO6, (d) M-rGO12, (e) M-rGO18, (f) M-rGO24 and the reference data for Fe_3_O_4_ of JCPDS no. 16–0629.

The presence of magnetite nanoparticles on the surface of rGO sheets was further investigated by XRD. Fig 2 displays the XRD peak profiles of GO and M-rGO nanocomposites. GO (Fig 2(A)) displayed a characteristic peak at 10.3° corresponding to (001). After the in situ chemical synthesis, GO and FeSO_4_ underwent a redox reaction, thereby forming M-rGO nanocomposites. In (Fig 2B–2F), the M-rGO nanocomposites (regardless of the stirring duration) showed six diffraction peaks at 2θ≈30.3°, 35.8°, 43.3°, 54.0°, 57.4°, and 63.0°, which corresponded to (220), (311), (400), (422), (511), and (440), respectively. These diffraction peaks were consistent with the database for the plane of reflection of face–centered cubic crystal magnetite (Fe_3_O_4_) (JCPDS no.19-0629). A small diffraction peak at 2θ≈18.4° appeared in M-rGO12 and M-rGO24, which was indexed to the (111) plane of magnetite [26]. No obvious change (including the intensity of the diffraction peaks) was observed in the XRD spectra of M-rGO even when the stirring duration was increased from 3 h to 24 h, suggesting that 3 h of stirring duration was sufficient for the formation of M-rGO in this study. No peak other than the magnetite peaks was observed in the XRD pattern of M-rGO, indicating the high purity of the produced M-rGO samples. In addition, the absence of the GO diffraction peak in the XRD spectra of the M-rGO nanocomposites was observed. This phenomenon suggested that during the synthesis of M-rGO nanocomposites, the oxygen-containing functional groups were removed from the GO surface because of the addition of NH_4_OH (reducing agent), resulting in the formation of the rGO sheets. Subsequently, magnetite nanoparticles were formed on the surface of the rGO sheets. The deposition of the magnetite nanoparticles can hinder the formation of van der Waals force and the π-π* stacking of the rGO sheet can therefore be prevented [33]. The diffraction peak for rGO was undetectable in the XRD pattern of M-rGO nanocomposites could also be due to another reason. According to Zhou et al. [34], no characteristic diffraction peak for rGO was detected in the XRD pattern because of the low amount and relatively low diffraction intensity of rGO. This hypothesis was supported by Sahu et al. [35], who declared that no significant peak was observed for rGO because of the weak crystallinity of carbon-based materials as compared with metal oxides (magnetite).The presence of magnetite nanoparticles and the disappearance of the GO characteristic peak indicated the successful synthesis of magnetite nanoparticles with rGO sheets through an in situ chemical synthesis. The average crystallite size of the magnetite nanoparticles formed on the surface of the rGO sheets can be calculated using the Debye Scherrer equation. The crystallite size of the magnetite nanoparticles of the M-rGO3, M-rGO6, M-rGO12, M-rGO18 and M-rGO24 are 14.43, 14.43, 14.75, 14.75 and 14.75 nm, respectively. The crystallite size of the magnetite nanoparticles formed by in situ chemical synthesis was consistent. A longer stirring duration corresponded to a slightly higher average crystallite size of magnetite nanoparticles. The XRD results validated that the magnetite nanoparticles were successfully attached on the rGO sheets, indicating that M-rGO nanocomposites were formed. In addition, the deposition of magnetite nanoparticles can suppress the agglomeration of rGO sheets and ensure the formation of uniformly sized magnetite nanoparticles with less agglomeration.

**Fig 3:**
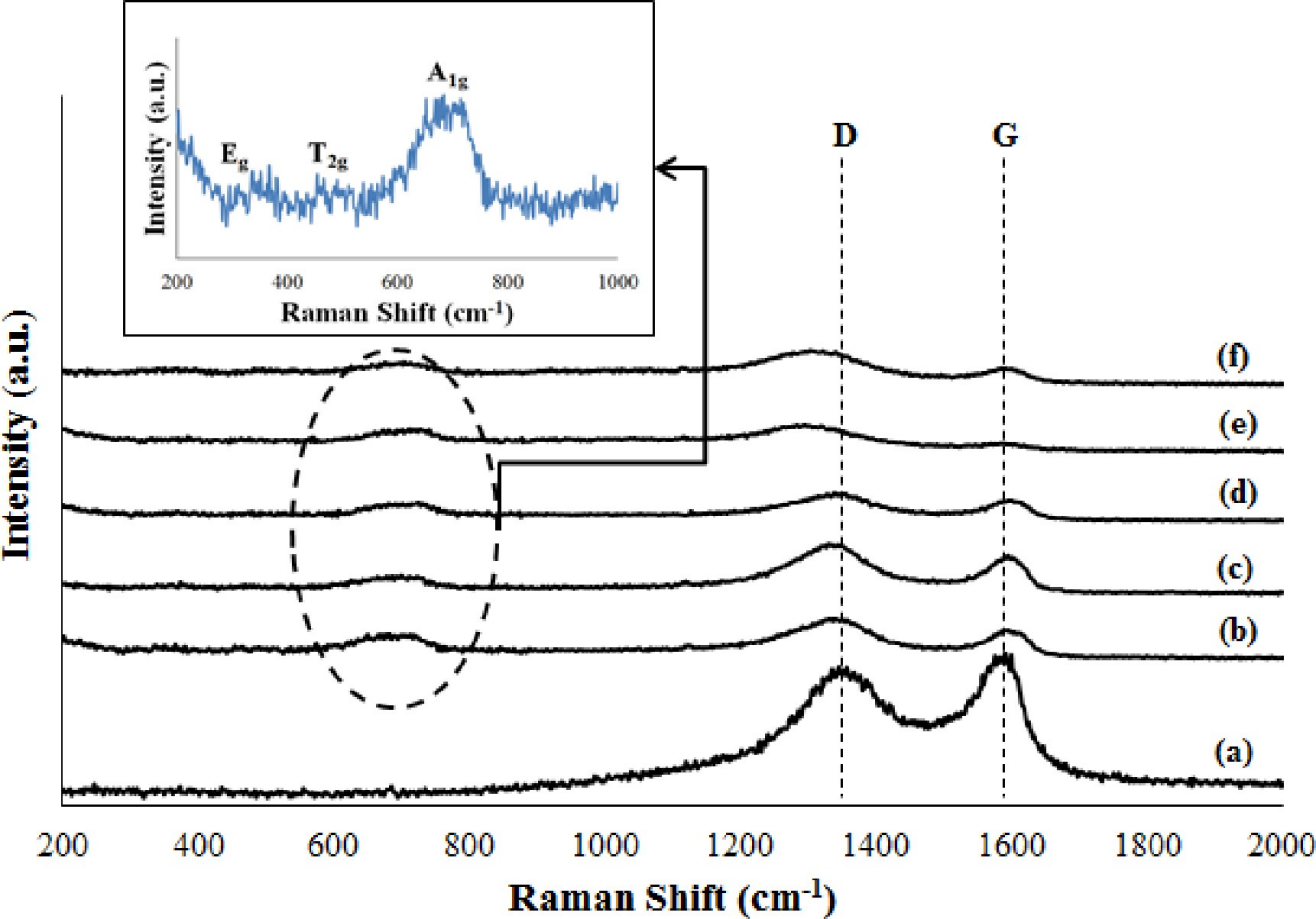
Raman spectra of (a) GO, (b) M-rGO3, (c) M-rGO6, (d) M-rGO12, (e) M-rGO18 and (f) M-rGO24. The inset shows the close view of the representative Raman spectrum (M-rGO3).

The formation of rGO sheets and magnetite nanoparticles in M-rGO nanocomposites was further confirmed by Raman spectroscopy, which is sensitive to changes in the carbon structure [36]. Fig 3 compares the Raman spectra of GO and M-rGO nanocomposites synthesized under different stirring durations. In Fig 3, Raman spectrum of GO displayed two main peaks, namely, the D (1346 cm^-1^) and G bands (1591 cm^-1^). After the in situ chemical synthesis, the Raman spectra of the as-synthesized M-rGO nanocomposites (regardless of the stirring duration) displayed additional characteristic peaks (aside from the main D and G bands), which was consistent with the findings by Sharif et al. [26]. The inset in Fig 3 shows the additional peaks located at 306, 485, and 668 cm^-1^, which were attributed to E_g_, T_2g_, and A_1g_, respectively [37]. These three peaks were the characteristic peaks of magnetite nanoparticles, thereby confirming the presence of magnetite nanoparticles in the M-rGO nanocomposites. The formation of rGO sheets in the M-rGO nanocomposites was further determined by measuring the intensity ratio of the D and G bands (I_D_/I_G_). The I_D_/I_G_ ratio is a measure of the defect density of rGO sheets and the extent of graphitization [38]. The I_D_/I_G_ ratio of GO and M-rGO nanocomposites are tabulated in Table 3. The I_D_/I_G_ ratio increased from 0.902 (GO) to 1.065 (M-rGO3) after the in situ chemical synthesis. This finding indicated the presence of sp^3^ defects within the sp^2^ carbon network upon the reduction of GO [39], thereby confirming the formation of rGO sheets in M-rGO nanocomposites. The I_D_/I_G_ ratio of M-rGO nanocomposites slightly decreased as the stirring duration was increased. This result suggested that M-rGO nanocomposites with 3 h of stirring duration saturated the magnetite nanoparticles on the surface of rGO sheets. M-rGO3 experienced numerous of defects, which were created during the reduction and attachment processes. The obtained value of the I_D_/I_G_ ratio agreed well with those reported in the literature [31, 38]. In addition, the broadness of the characteristic peaks (D and G bands) and the higher intensity of the D band than that of G band in the Raman spectra of the M-rGO nanocomposites suggested that more defects were generated in the synthesis process, thereby confirming the effective reduction of GO and the successful attachment of magnetite on the rGO sheets. The D band slightly shifted in the Raman spectra of M-rGO18 and M-rGO24. According to Bharath et al. [40], a shift in the D band and an increased intensity ratio of M-rGO nanocomposites as compared with GO may be attributed to the decrease in the average size of the sp^2^ domain. Therefore, M-rGO might have been highly disordered, and the magnetite nanoparticles might have been randomly arranged on the surface of rGO sheets.

**Table 3:** Degree of disorder (I_D_/I_G_) of GO and M-rGO.

Sample	I_D_/I_G_ ratio
GO	0.902
M-rGO3	1.065
M-rGO6	1.049
M-rGO12	1.026
M-rGO18	1.045
M-rGO24	1.039

**Fig 4:**
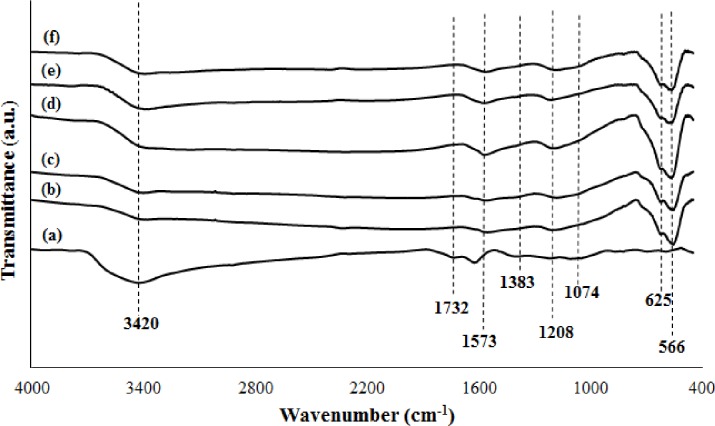
FTIR spectra of (a) GO, (b) M-rGO3, (c) M-rGO6, (d) M-rGO12, (e) M-rGO18 and (f) M-rGO24.

FTIR spectroscopy analysis was used to further study the surface chemistry, bonding nature, and chemical structure of GO and M-rGO nanocomposites [40]. In this study, FTIR spectroscopy analysis was performed to prove the interaction between magnetite nanoparticles and rGO sheets [41]. Fig 4 shows the FTIR spectra of the GO and M-rGO nanocomposites. The FTIR spectrum of GO (Fig 4(A)) had six characteristic peaks, namely, CO–O–CO at 1074 cm^-1^, epoxy C–O at 1219 cm^-1^, carbonyl C = O at 1383 cm^-1^, aromatic C = C stretching at 1624 cm^-1^, carboxyl O = C–OH at 1732 cm^-1^, and hydroxyl (–OH) at 3420 cm^-1^. The presences of oxygenated functional groups at GO indicated that the graphite underwent oxidation. (Fig 4B–4F) show the FTIR spectra for M-rGO nanocomposites production with 3, 6, 12, 18, and 24 h of stirring duration. In general, the FTIR spectra of M-rGO nanocomposites in (Fig 4B–4F) possessed similar characteristic peaks located at approximately 3420, 1573, 1208, 625, and 566 cm^-1^, which were ascribed to the broad–OH, C = C stretching peak, epoxy C–O, and Fe–O lattice vibrations, respectively. Table 4 presents a summary of the functional groups obtained through the FTIR analysis of GO and M-rGO nanocomposites. The oxygenated functional groups of GO, such as the carboxylic acid (O = C–OH) and carbonyl groups (C = O), were no longer detected in the FTIR spectrum of M-rGO nanocomposites (regardless of the stirring duration). In addition, the intensity of the peaks of hydroxyl group (-OH) and epoxy (C–O) groups in the FTIR spectra of M-rGO nanocomposites were lower than those of GO. All of these happens was due to the removal of the oxygen-containing functional groups from the GO surface during in situ chemical synthesis to form rGO sheets for the attachment of magnetite nanoparticles. According to Sahu et al. [35], the presence of the C = C and C–O characteristic peaks at 1573 and 1208 cm^-1^, respectively, confirmed the formation of rGO in the M-rGO nanocomposites. The red shifted of the transmittance peak C–O at 1208 cm^-1^ occurred because of the Fe–O–C vibration, which confirmed the interaction between magnetite and rGO sheets [35]. The presence of a Fe–O lattice vibration at 625 and 566 cm^-1^ in the FTIR spectra of M-rGO nanocomposites proved that magnetite nanoparticles were successfully loaded on the surface of rGO sheets [31]. A noticeable intensity of the peak of Fe–O lattice vibration displayed 3 hours of stirring duration is enough to form M-rGO nanocomposites.

**Table 4:** FTIR analysis of GO and M-rGO.

Peak Value (cm^-1^)	Functional Groups	GO	M-rGO
3420	Hydroxyl–OH	Yes	Yes
1732	Carboxylic acids O = C–OH	Yes	N/A
1624	Aromatic C = C stretching	Yes	Yes
1573			
1383	Carbonyl C = O	Yes	N/A
1219	Epoxy C–O	Yes	Yes
1208			
1074	Anhydride group CO–O–CO	Yes	N/A
625	Fe–O	N/A	Yes
566	Fe–O	N/A	Yes

**Fig 5:**
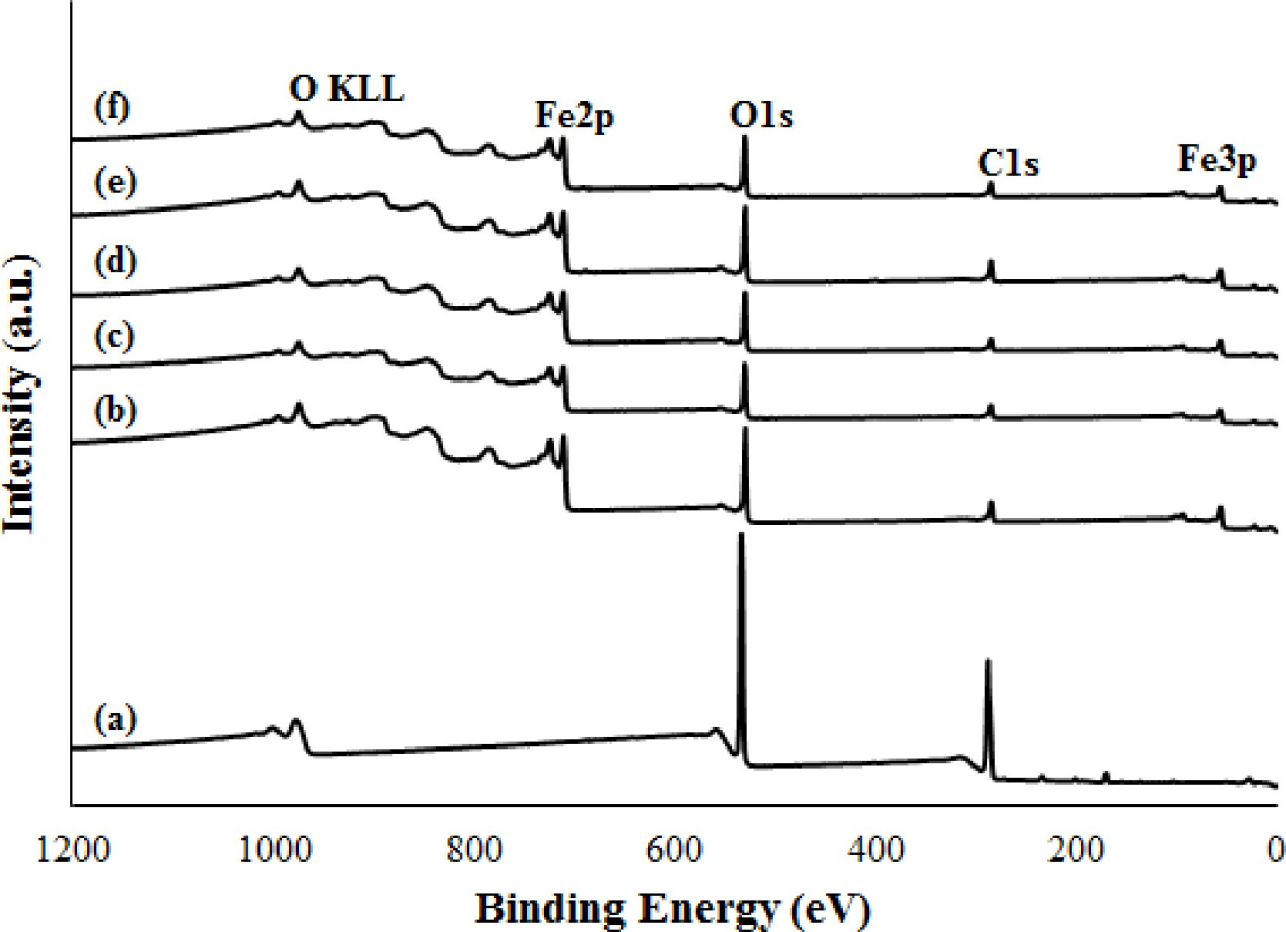
XPS survey spectra of (a) GO, (b) M-rGO3, (c) M-rGO6, (d) M-rGO12, (e) M-rGO18 and (f) M-rGO24.

XPS analysis was performed to obtain further information regarding the surface composition and the elemental chemical oxidation states of GO and M-rGO nanocomposites. Fig 5 shows the XPS survey spectra of GO and M-rGO nanocomposites. As shown in Fig 5(A), GO shows peaks at around 284 and 530 eV, which are correspond to the C 1s and O 1s core spectra, respectively. Meanwhile, the M-rGO nanocomposites in (Fig 5B–5F) exhibit extra peaks, such as Fe 2p and Fe 3p, which confirms the formation of magnetite nanoparticles [41]. The relative intensity of the characteristic peaks (C 1s, O 1s, and Fe 2p) was estimated from the area under the curves by integrating the peak areas [42]. The intensity peaks for the C 1s and O 1s of GO were initially about 62.39 At.% and 37.61 At.%, respectively. However, after in situ chemical synthesis was completed, the M-rGO nanocomposites achieved relative intensities of about 31–36, 42–45, and 21–26 At.%, which correspond to C 1s, O 1s, and Fe 2p, respectively. The substantial reduction of the relative intensity of C 1s, the increments of the relative intensity of O 1s that might have originated from magnetite nanoparticles and the emergence of the Fe 2p characteristic peak suggest that the GO was successfully reduced to rGO, whereas the magnetite nanoparticles attached onto the surface of the rGO sheets. This phenomenon was consistent with the EDX analysis from FESEM.

**Fig 6:**
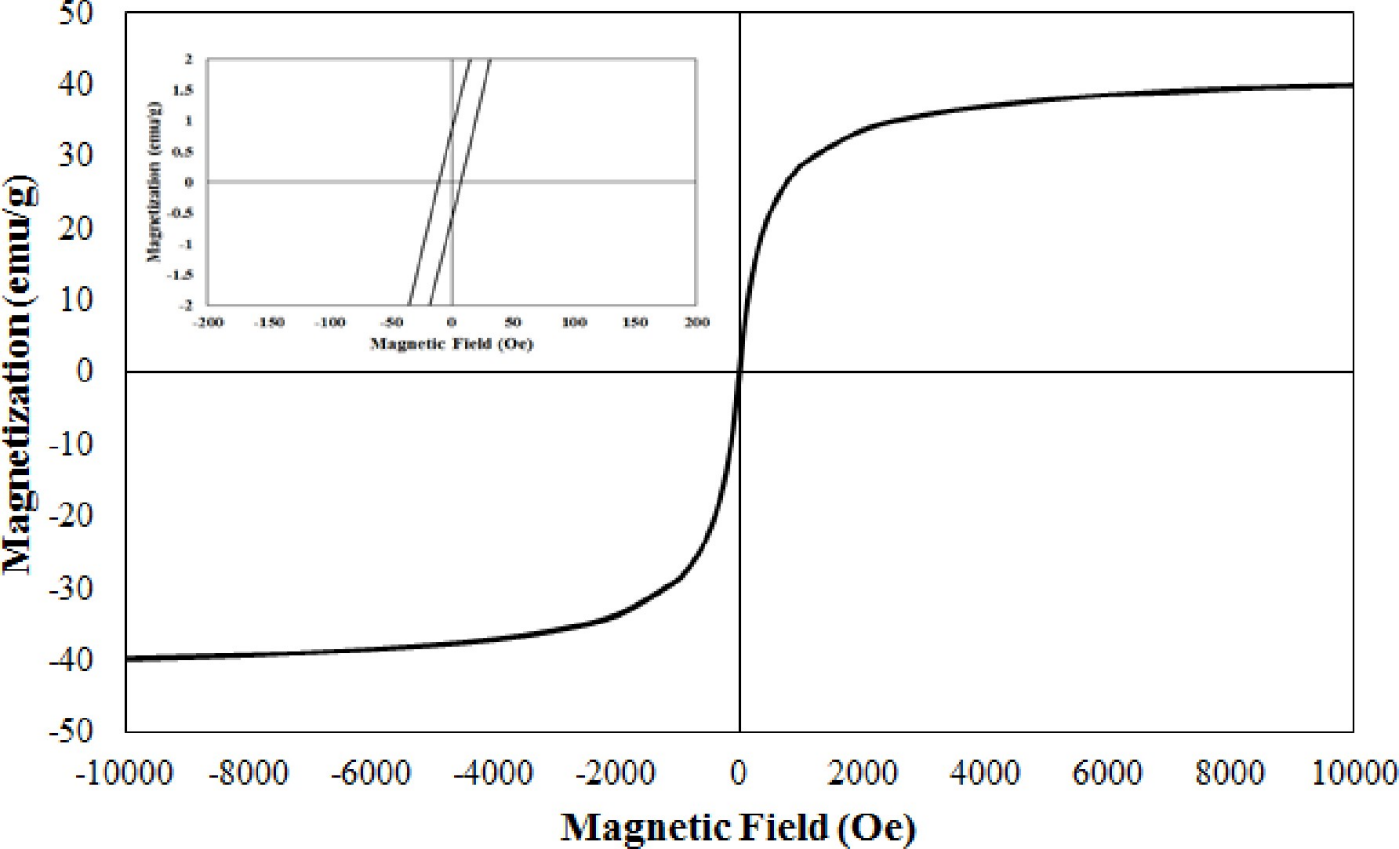
Room temperature magnetization curve and a close view of the magnetization curve (inset) of M-rGO3.

The magnetic behavior of M-rGO nanocomposites was studied using a VSM. M-rGO3 was selected as a representative sample for VSM analysis. The magnetic properties of M-rGO3 were investigated at room temperature (298 K) and presented in Fig 6. The magnetic hysteresis curve of M-rGO3 exhibited a typical sigmoid-like (S-like) curve. The magnetic remanence and magnetic coercivity of M-rGO3 were 0.716 emu/g and 8.88 Oe, respectively. The low remanence and coercivity revealed the absence of magnetization upon the removal of the external magnetic field and indicated the superparamagnetic behavior of M-rGO3 nanocomposites [39]. The saturation magnetization (M_s_) of the M-rGO3 nanocomposites was 40.8 emu/g, which was lower than the reported M_s_ value for the bulk magnetite (92.8 emu/g) [43] likely because of the presence of rGO sheets and the small size of magnetite nanoparticles [39]. The rGO sheets may act as a diamagnetic material that can reduce the saturation magnetization of M-rGO nanocomposites [41]. Saturation magnetization decreases as the size of magnetite nanoparticles decreases [44]. In the current work, the size of the magnetite nanoparticles that attached onto the surface of the rGO sheets was approximately 15 nm (from SEM analysis). The M_s_ obtained for this sample was 40.8 emu/g. In comparison with the values described in previous works [45, 46], the obtained M_s_ in our study was sufficient and suitable for magnetic separation and targeting.

### Demulsification of the crude O/W emulsion

In this study, M-rGO3 nanocomposites were selected as demulsifier to perform a demulsification test on crude O/W emulsion. This consideration was because of the impressive results obtained in previous characterization tests as follows:

1. The size of M-rGO3 nanocomposites (approximately 15.91±2.74 nm) obtained through FESEM analysis. FESEM analysis showed that the adequate amount of uniform-sized magnetite nanoparticles were loaded on the surface of the rGO sheets.
2. In the Raman analysis, the M-rGO3 achieved the highest I_D_/I_G_ ratio (I_D_/I_G_ = 1.065) relative to those of others, indicating that more magnetite nanoparticles were attached onto the rGO sheets.
3. The magnetite nanoparticles had an M_s_ value of 40.8 emu/g, which allowed magnetic separation and targeting in demulsification.
4. Moreover, a short stirring duration (only 3 h) was adequate for synthesizing the M-rGO nanocomposites. Thus, the procedure was time and energy saving.

**Fig 7:**
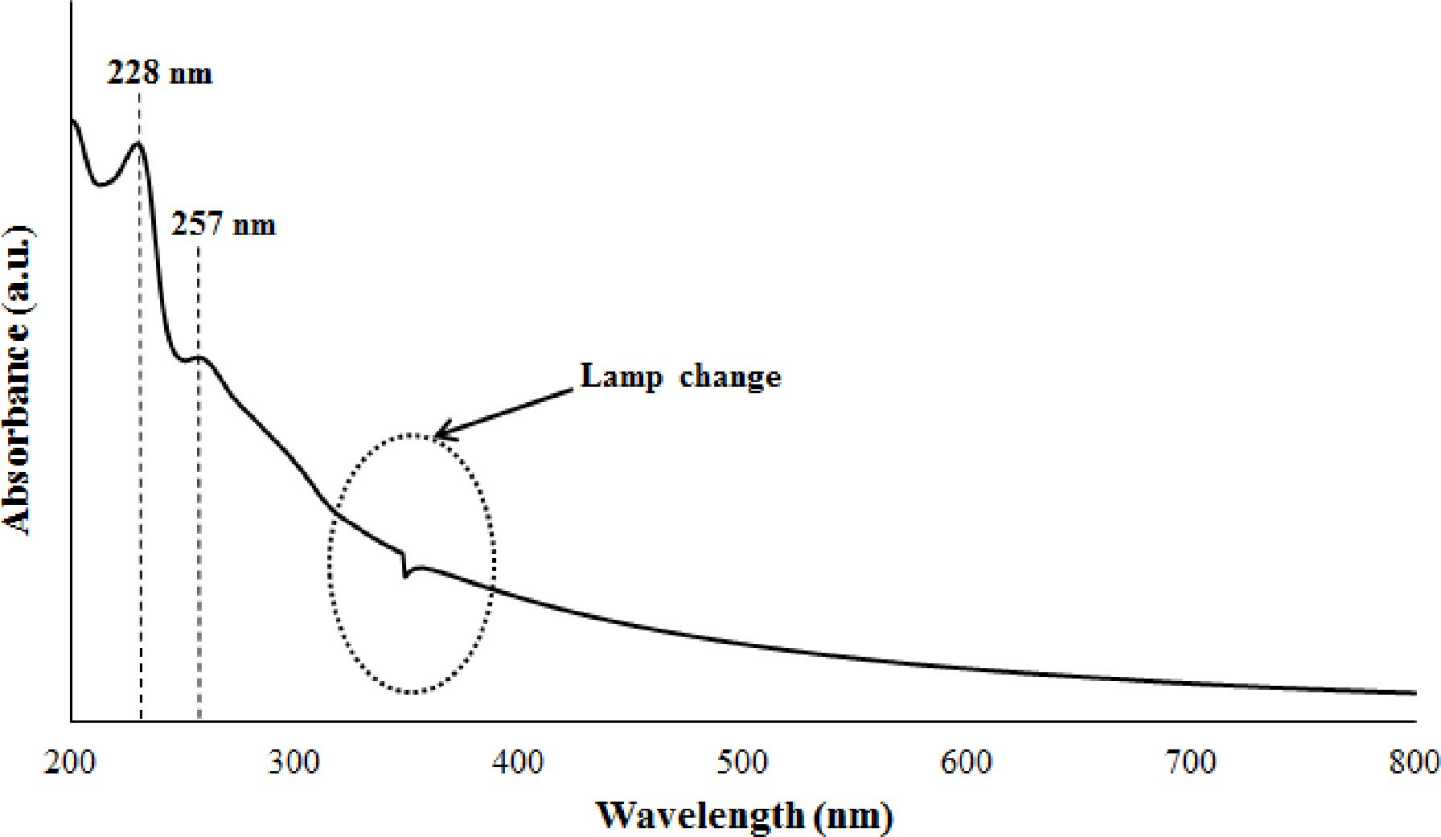
UV-Vis spectrum for crude O/W emulsion (264 mg L^-1^).

To have a better understanding on demulsification process, few works have been done prior to demulsification test. First of all, UV-Vis was performed to determine the characteristic absorption peaks of crude O/W emulsion. The wavelength of the adsorption peaks can also serve as a standard guideline for determining the oil content in residue water after a demulsification test is performed [47]. Fig 7 shows the UV-Vis spectrum of the crude O/W emulsion at a concentration of 264 mg L^-1^. In Fig 7, two peaks were displayed in the UV-Vis spectrum of the crude O/W emulsion: 228 nm corresponding to benzene compounds and 257 nm corresponding to naphthenic compounds [48].

**Fig 8:**
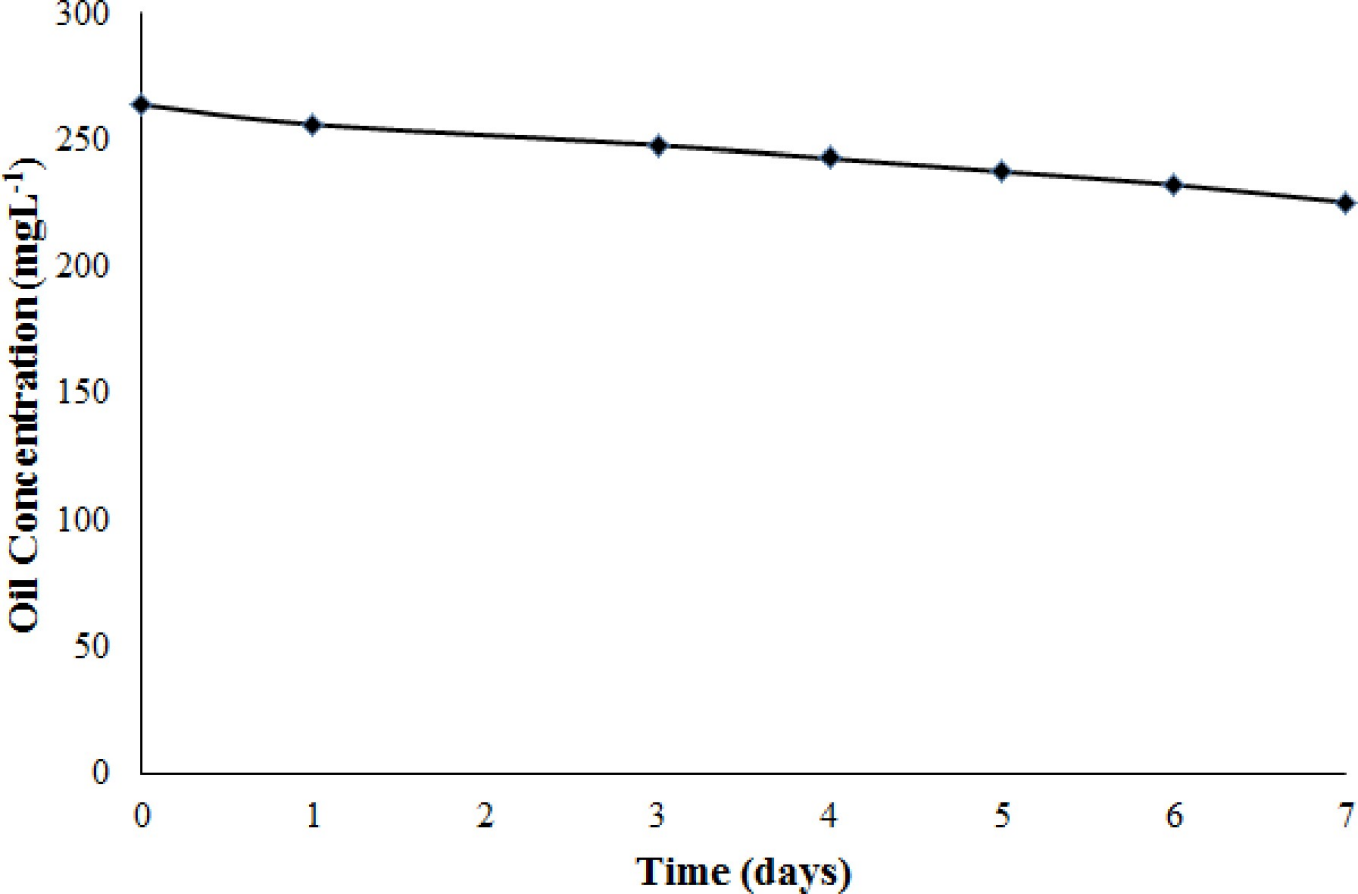
Stability of crude O/W emulsion over the time.

Next, a stability test (Fig 8) was conducted to investigate the stability of crude O/W emulsion in the presence of Tween 60 over time (days). In this study, Tween 60 was selected and used as a surfactant to stabilize the crude O/W emulsion. The involvement of Tween solution in the preparation of O/W emulsion is similar to those in reported literature [15, 27, 49]. Fig 8 shows the crude O/W emulsions were stable for 7 days. On day 7, the oil concentrations of the crude O/W emulsion was 225.50 mg L^-1^ (*E*_D_ = 14.58%). Notably, only a small decrement in oil concentration was observed in crude O/W emulsions relative to the initial oil concentration (264 mg L^-1^), suggesting that Tween 60 plays a stabilizing role in crude O/W emulsions.

**Fig 9:**
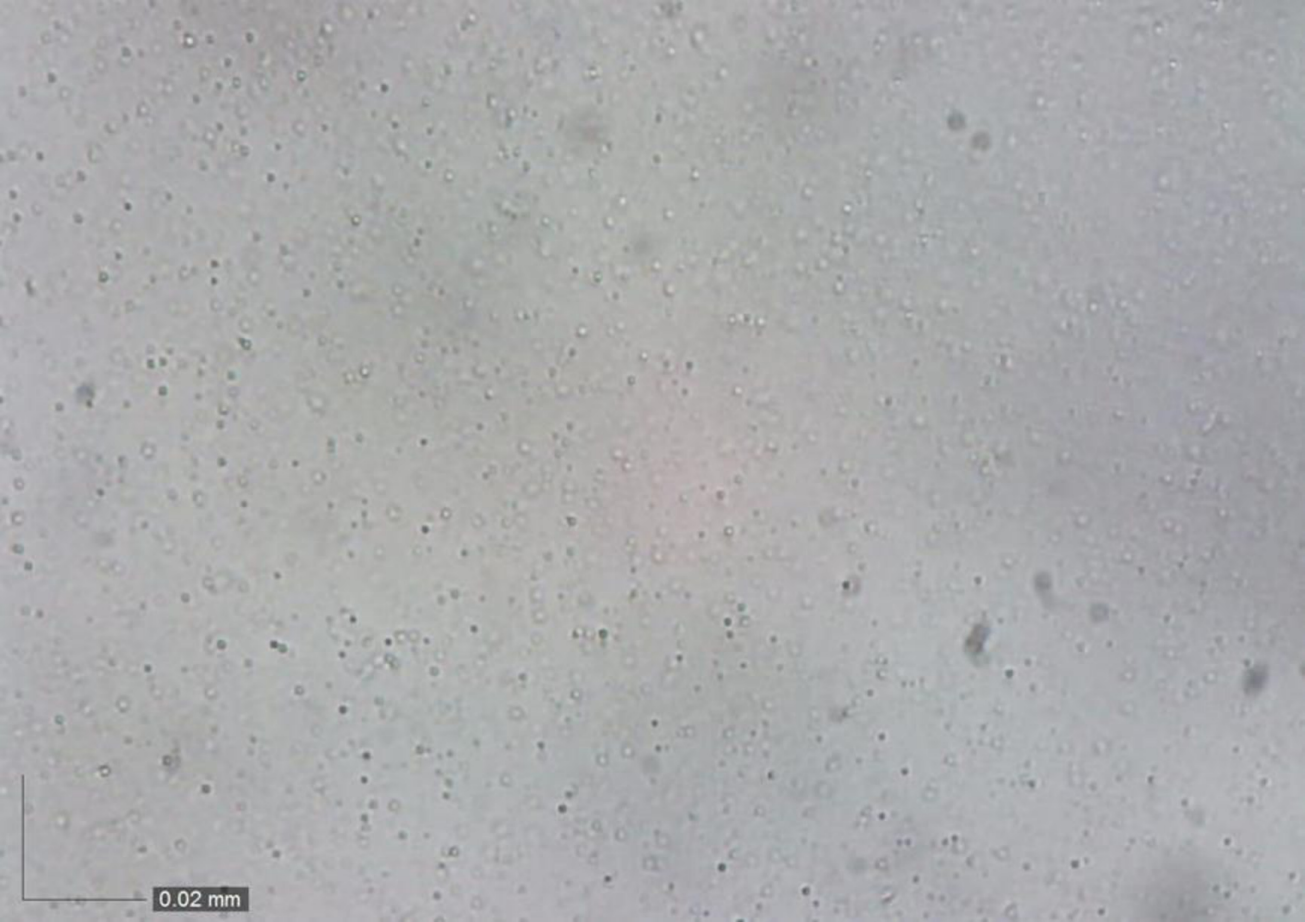
Optical microscopy image of crude O/W of the 7^th^ day.

Lastly, the stability of the crude O/W emulsion was further investigated through optical microscopy. Micrometer-sized oil droplets were observed. In Fig 9, the oil droplets measuring 0.5–2 μm in diameter were scattered throughout the entire view of the image. No flocculation of oil droplets was observed, suggesting that the oil droplets were homogeneously dispersed in the water phase. This observation was consistent with that described in previous studies [46, 50]. An emulsion was successfully formed and stabilized with the aid of Tween 60. Dynamic light scattering (DLS) was also applied to confirm the droplet size of the crude O/W emulsion. DLS measurements revealed that the crude O/W emulsion in deionized water had a mean oil droplet size of 1.67 μm, which agreed with the optical microscopy result.

**Fig 10:**
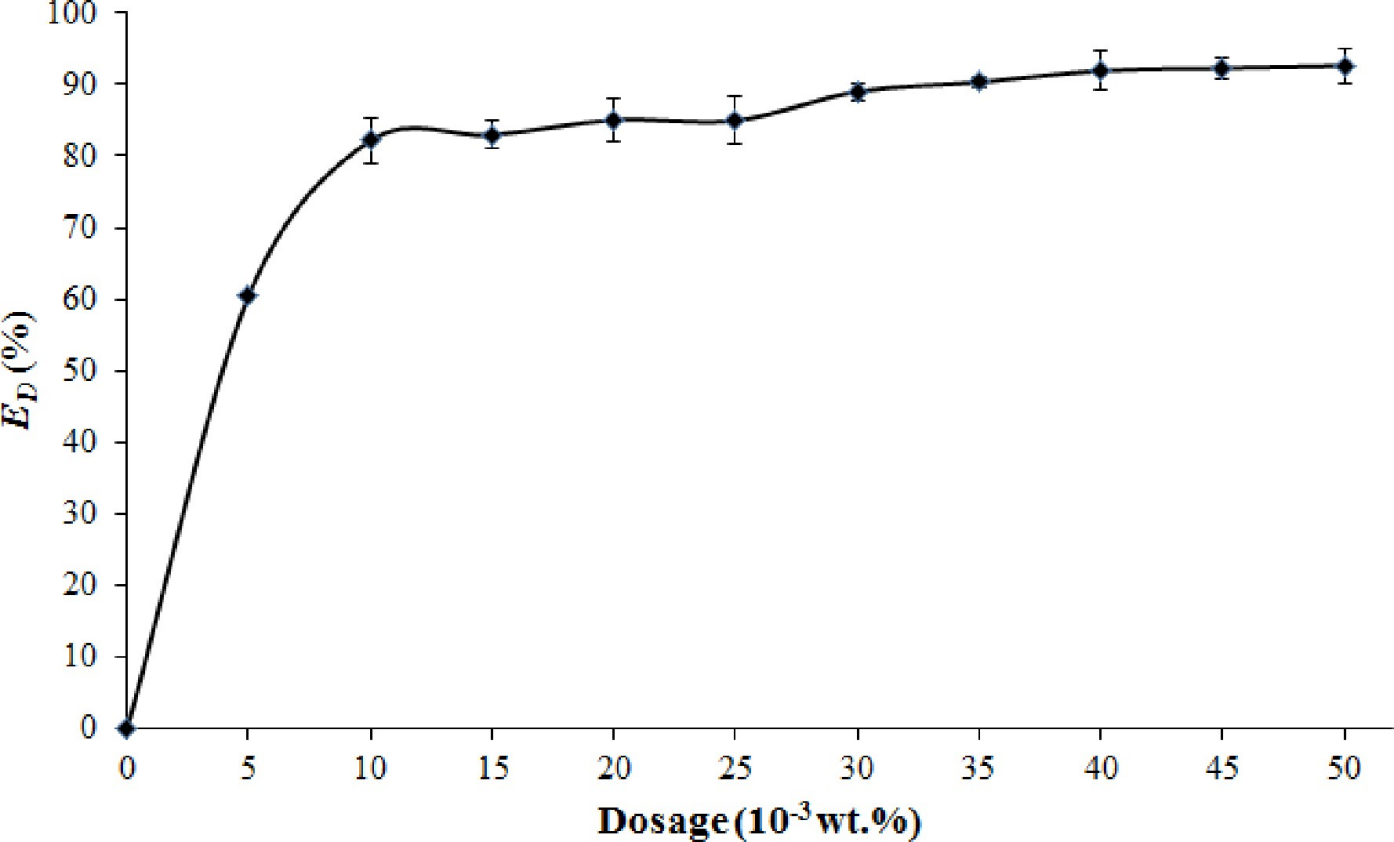
Effect of M-rGO nanocomposites dosage on *E*_D_ for crude O/W emulsion.

In demulsification test, the proper dosage of demulsifier for the demulsification of crude O/W emulsion should be determined. This is due to a very low dosage of demulsifier leaves the emulsion unchanged, but an excessive amount of demulsifier may produce a stable emulsion [51]. Thus, the effect of demulsifier dosage on demulsification efficiency (*E*_D_) or oil removal rate was investigated in this study. Different dosages of M-rGO nanocomposites in the range of 0.005 wt.% to 0.050 wt.% were applied to crude O/W emulsion. The settling time was kept constant at 2 min at room temperature. A control sample without an added demulsifier was also introduced in this study. Fig 10 illustrates the effect of M-rGO nanocomposite dosage on the *E*_D_. In the control sample, no oil droplet was removed (*E*_D_ = 0%) from the crude O/W emulsion. However, the removal of oil droplets was happened once the M-rGO nanocomposites (demulsifier) were added to the emulsion (change in *E*_D_). When the M-rGO nanocomposites with a dosage of 0.005 wt.% were added to the emulsion, the *E*_D_ of the crude O/W emulsions increased abruptly to about 60%. In Fig 10, the M-rGO nanocomposites could thoroughly demulsify the crude O/W emulsion. Notably, the increased demulsifier dosage resulted in an increase in *E*_D_. When the M-rGO nanocomposite dosage increased from 0.010 wt.% to 0.030 wt.%, the *E*_D_ of M-rGO nanocomposites in crude O/W emulsion increased from 82.13% to 89.00%. However, the *E*_D_ did not obviously change when the M-rGO nanocomposite dosage continuously increased from 0.035 wt.% to 0.050 wt.%. For example, in the crude O/W emulsion, the *E*_D_ reached 92.26% as the M-rGO nanocomposite with a dosage of 0.045 wt.% was added to the emulsion. Further increasing the M-rGO nanocomposite dosage to 0.050 wt.% slightly increased the *E*_D_ (about 0.35%). Overall, these findings suggested that the M-rGO nanocomposites could be used as an effective demulsifier to break the crude O/W emulsion.

**Fig 11:**
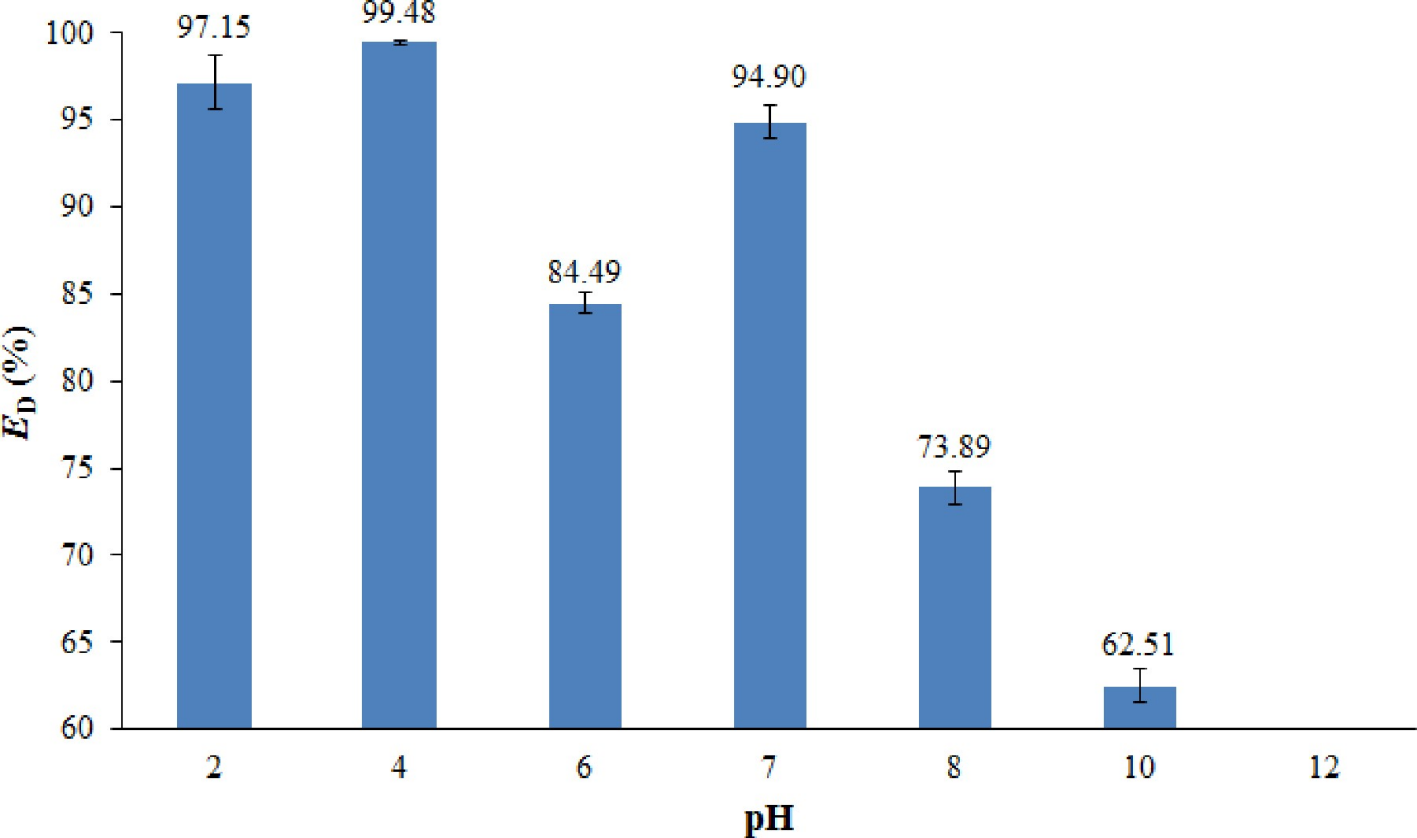
Effect of the crude O/W emulsion pH on *E*_D_.

Besides, the pH of the crude O/W emulsion is also an important factor that should be considered in demulsification tests. Wang et al. [52] demonstrated that a change in the pH of crude O/W emulsion may influence the flocculation/aggregation of oil droplets and the demulsification performance. Therefore, in this study, the effect of crude O/W emulsion pH was investigated by varying the pH of the crude O/W emulsion (264 mg L^-1^) at a range of 2–12 by using 0.5 M HCl and 0.5 M NaOH solutions. Fig 11 shows the effects of the pH of crude O/W emulsion on the *E*_D_. The dosages of the M-rGO nanocomposites (0.05 wt.%), the initial oil concentration of crude O/W emulsion (264 mg L^-1^), and the temperature condition of the demulsification test (25°C) were kept constant throughout the experiment. In a crude O/W emulsion system (Fig 11), the *E*_D_ under acidic and neutral conditions was approximately 85% and above. Conversely, the *E*_D_ decreased under alkaline conditions, especially strong alkaline conditions (pH = 10). This phenomenon was similar to the observation of Wang et al. [53]. Under acidic conditions (pH < 7), a high *E*_D_ was recorded because of a high degree of electrostatic attraction between the M-rGO nanocomposites and the oil droplets in emulsion [3]. Under alkaline conditions, low *E*_D_ was obtained as the surface charge of the M-rGO nanocomposites became negative and consequently caused a repelling interaction between the M-rGO nanocomposites and emulsified oil droplets [3]. The zeta potential of the M-rGO nanocomposites (will be discussed in next section) can be used to explain the electrostatic attraction/repulsion between the M-rGO nanocomposites and the emulsified oil droplets. In this study, crude O/W emulsion had optimal pH of 4 (with *E*_D_ = 99.48%), favoring the best demulsification performance. Thus, this emulsion pH value was used in the subsequent recycle test.

### Plausible mechanism of demulsification

**Fig 12:**
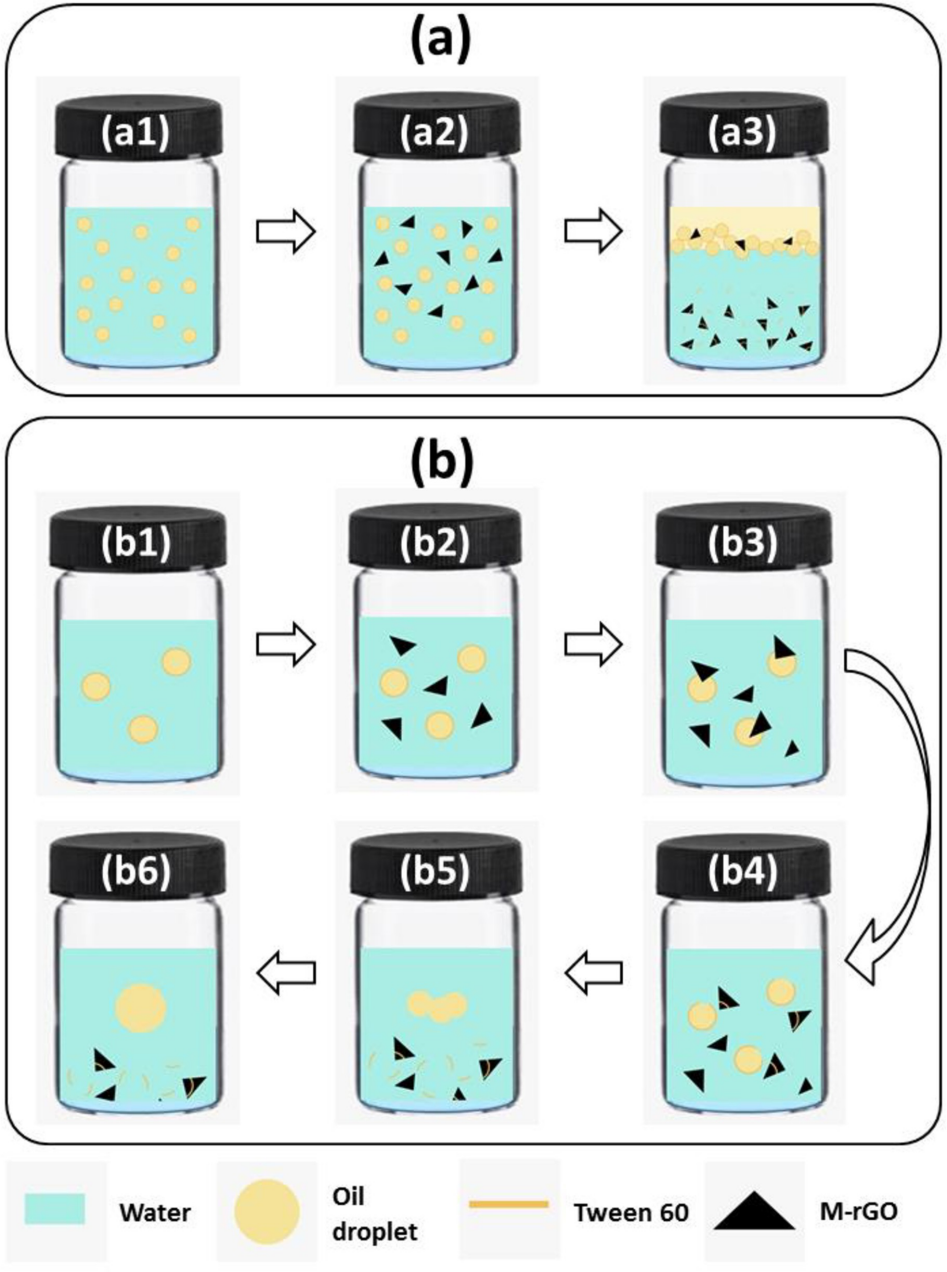
Schematic illustration of (a) the demulsification process and (b) the possible demulsification mechanism.

A plausible demulsification mechanism was proposed on the basis of experimental results and shown in Fig 12. Tapis crude oil and water are two immiscible liquids that cannot disperse well in each other in the absence of a surface-active agent. In this study, Tween 60 (surface active agent) was added to form a crude O/W emulsion by reducing the tension between the oil phase and the water phase. Tween 60 is composed of a hydrophilic head and a hydrophobic tail oriented toward the water and oil phases, respectively, thereby forming a stable crude O/W emulsion (Fig 12(A1) and 12(B1)). After M-rGO powder was added to crude O/W emulsion (Fig 12(A2) and 12(B2)), M-rGO nanocomposites entered the O/W interface upon shaking (Fig 12(B3)) and subsequently breaking up the interfacial film formed by Tween 60 (Fig 12(B4)). After the interfacial film was disrupted, small oil droplets underwent flocculation (Fig 12(B5)) and subsequently coalesced into large oil droplets (Fig 12(B6)). After a certain time, the coalescence oil droplets (lower in density than water) floated on the top of the solution (Fig 12(A3)), whereas the oil droplets tagged on the surface of M-rGO nanocomposites were rapidly removed from the water phase under the applied external magnetic field. The increase in the dosage of the M-rGO nanocomposites (demulsifier) caused the interaction of Tween 60 (emulsifier) and crude O/W emulsion molecules to reach equilibrium at the interface, decreased the strength of the interfacial film, and caused the oil droplets to coalesce. This effect might result in the further separation of oil droplets from the emulsion (high *E*_D_). However, if an excessive dosage of demulsifier is applied to the crude O/W emulsion, demulsifier molecules may form a new interfacial film and stabilize the emulsion [54].

**Fig 13:**
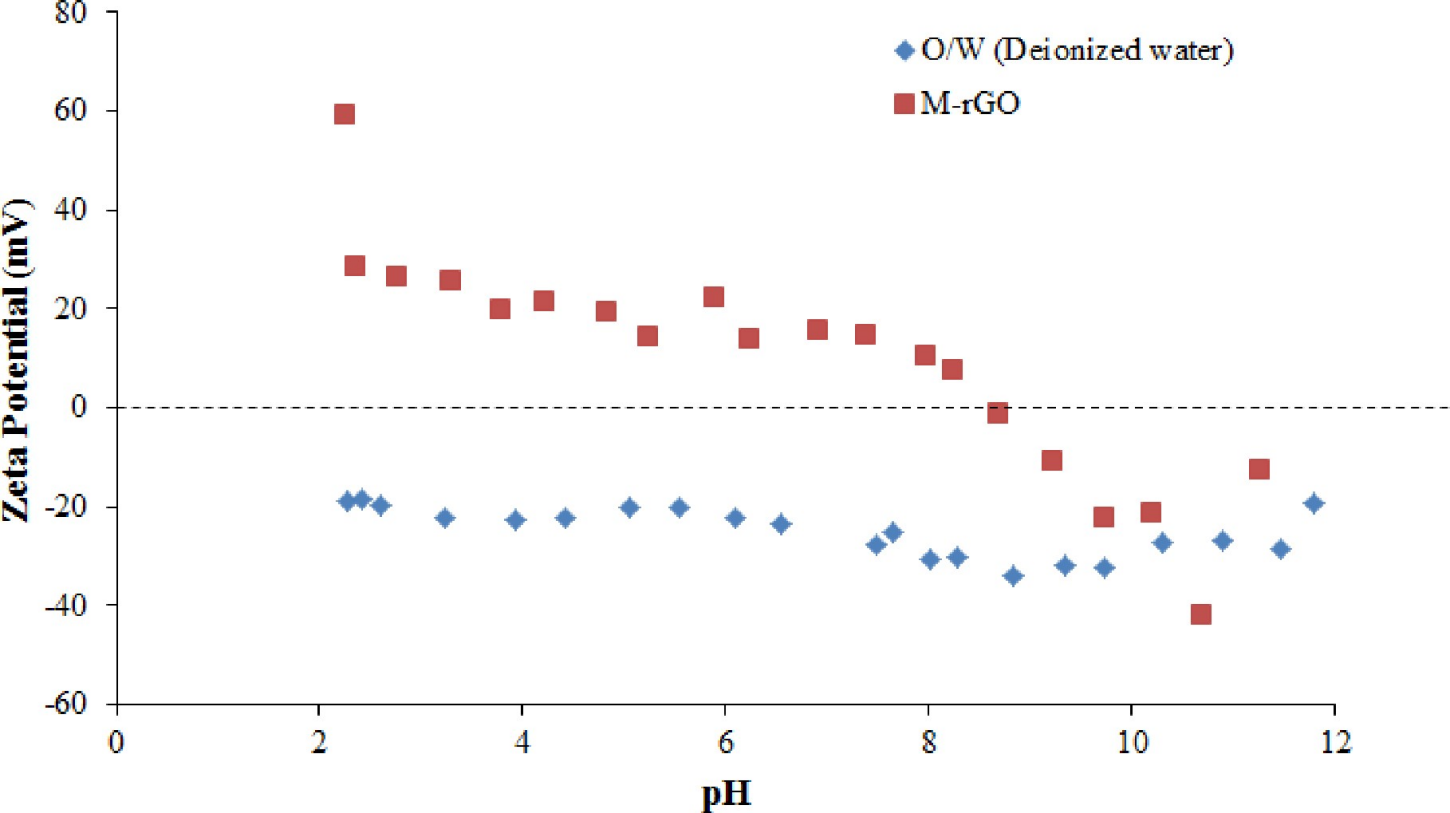
Zeta potential of M-rGO nanocomposites and crude O/W emulsion at various pH values.

To obtain further insights into the demulsification efficiency, especially the effect of pH, zeta potentials of the crude O/W emulsions and M-rGO nanocomposites were measured. The electrostatic interaction between the M-rGO nanocomposites and the emulsified oil droplets could play an important role in the demulsification test because surface charges vary with pH [4]. Fig 13 displays the zeta potentials of the crude O/W emulsion in comparison with the zeta potential of the M-rGO nanocomposites. The zeta potentials of the M-rGO nanocomposites revealed a classical “S” shape, that is, positive at pH lower than 8 and negative at pH higher than 8. Thus, the zeta potential of the M-rGO nanocomposites decreased as solution pH increased. This phenomenon is similar to those observed by Lü et al. [4]. Meanwhile, the crude O/W emulsion was negatively charged at all pH tested. The difference in the surface charge of the M-rGO nanocomposites and the emulsified oil droplets tended to promote the demulsification efficiency via the electrostatic attraction of the M-rGO nanocomposites toward the emulsified oil droplets. For example, in the crude O/W emulsion system at pH 4 (acidic condition), M-rGO nanocomposites were positively charged (+21.47 mV), whereas the emulsified oil droplets was negatively charged (−22.30 mV). The opposite charge between the components causes the M-rGO nanocomposites to enter the oil–water interface easily through electrostatic attraction and subsequently promotes the flocculation and coalescence of oil droplets [4]. This phenomenon was similar to the demulsification test of crude O/W emulsion under a neutral condition (pH = 7) in which the M-rGO nanocomposites were positively charged (+14.57 mV) and the emulsified oil droplets were negatively charged (−27.61 mV). Thus, the demulsification efficiency under acidic and neutral conditions was impressive, that is, more than 85%. However, at a higher pH (pH > 8), the M-rGO nanocomposites became negatively charged. The emulsified oil droplets in crude O/W emulsion also remained negatively charged. The identical charge of the M-rGO nanocomposites and emulsified oil droplets caused the electrostatic repulsion to occur; subsequently, the M-rGO nanocomposites were unable to attach to the emulsified oil droplets. Thus, the *E*_D_ of M-rGO nanocomposite in crude O/W emulsion decreased drastically under alkaline conditions (pH > 8). These results indicated that M-rGO nanocomposites were pH sensitive, and their demulsification performance was dependent on the surface charge of the emulsified oil droplets at various pH values.

### Recyclability of demulsifier

**Fig 14:**
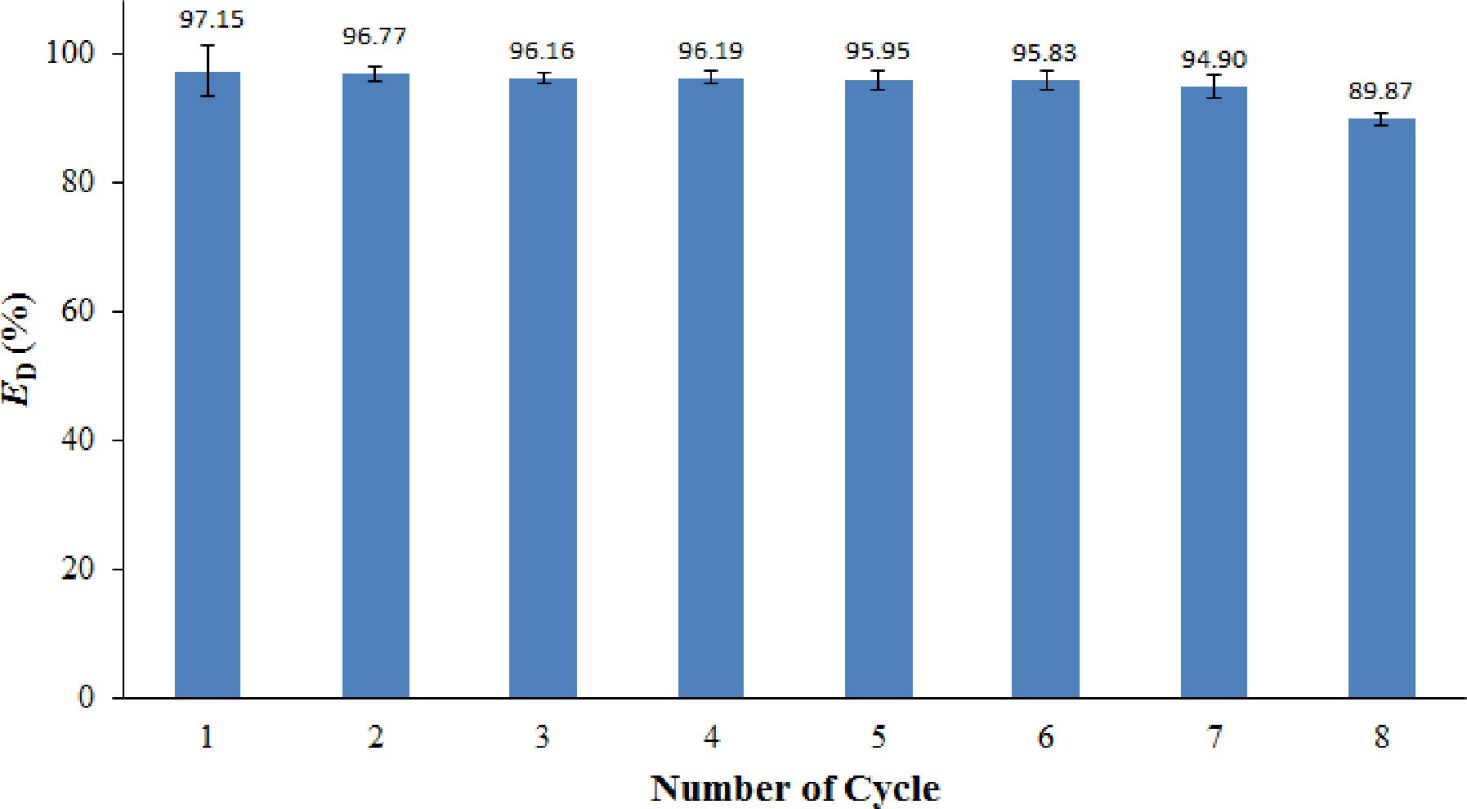
*E*_D_ of M-rGO nanocomposites in crude O/W emulsion during various cycles.

Unlike the conventional chemical demulsifiers, M-rGO nanocomposites can be recycled and reused after demulsification because of their superparamagnetic feature [55]. The recyclability of M-rGO nanocomposites in crude O/W emulsion was investigated in this study. For the crude O/W emulsion, the recycling test was performed at pH 4. The selection of pH of crude O/W emulsion was based on the best performance of M-rGO nanocomposites in such a pH environment as discussed in the previous section. Fig 14 shows the recyclability of the M-rGO nanocomposites in crude O/W emulsion for 8 cycles. In the crude O/W emulsion (Fig 14), the M-rGO nanocomposites in the first 7 cycles did not significantly change in terms of *E*_D_. However, after the 7th cycle, *E*_D_ decreased to 89.87%. The small amount of oil droplets tagged on the surface of M-rGO nanocomposites could not be easily removed by ethanol during the washing process, especially after several cycles, thus causing a gradual decrease in *E*_D_ during recycling. This phenomenon was in good agreement with the finding proposed by Liu and his co-workers [46]. Overall, the M-rGO nanocomposites still showed an excellent demulsification performance and good recyclability for crude O/W emulsion in this study. The recycle and reuse of M-rGO nanocomposites (demulsifier) consequently reduced the chemical cost in practical applications. The side effect of demulsifiers on the environment was greatly reduced because the M-rGO nanocomposites could be recycled by applying an external magnetic field.

## Conclusion

In this study, M-rGO nanocomposites were selected as a magnetic demulsifier and applied to treat crude O/W emulsion that is stabilized by Tween 60 (surfactant). M-rGO nanocomposites were successfully synthesized via in situ chemical synthesis at room temperature. The effect of stirring duration on the formation of M-rGO nanocomposites was characterized by FESEM, XRD, Raman, FTIR and XPS. Results showed that M-rGO nanocomposites were successfully synthesized via in situ chemical synthesis at room temperature. The efficiency of M-rGO nanocomposites in demulsifying the surfactant stabilized crude O/W emulsion was also evaluated. M-rGO nanocomposites with 3 h of stirring duration (M-rGO3) were selected as a magnetic demulsifier due to the small and uniform size of magnetite nanoparticles evenly distributed on the rGO sheet. M-rGO3 also exhibited superparamagnetic behaviour at room temperature, as indicated by VSM analysis. The demulsification efficiency increased with an increase in the demulsifier dosage. M-rGO nanocomposites had good demulsification efficiency under acidic and neutral conditions. In addition, a plausible mechanism for the demulsification was proposed in this study. M-rGO nanocomposites entered the oil-water interface upon shaking and then broke up the interfacial film formed by Tween 60. The emulsified oil droplets underwent flocculation and coalescence, and finally separated from water with the aid of an external magnetic field. Besides, results also shown that the difference in the surface charges of M-rGO nanocomposites and emulsified oil droplets promoted the demulsification efficiency via electrostatic attraction. From recycle test, M-rGO nanocomposites can be reused up to 6 cycles without a considerable loss in demulsification efficiency, thereby highlighting their good reusability. Indeed, the findings in this work provide a new perspective to develop simple, effective, eco-friendly and recyclable demulsifier for surfactant stabilized crude O/W emulsion.
