# Correction: Magnetically recoverable magnetite-reduced graphene oxide as a demulsifier for surfactant stabilized crude oil-in-water emulsion

**DOI:** 10.1371/journal.pone.0298915

**Published:** 2024-02-12

**Authors:** Xin Hui Yau, Cheng Seong Khe, Mohamed Shuaib Mohamed Saheed, Chin Wei Lai, Kok Yeow You, Wai Kian Tan

There are errors in the Funding section. The correct Funding statement is: This research is supported financially by the Fundamental Research Grant Scheme (FRGS/1/ 2019/STG07/UTP/02/6) and the Yayasan Universiti Teknologi PETRONAS (YUTP) Grant (YUTP 0153AA-E40). The funders had no role in study design, data collection and analysis, decision to publish, or preparation of the manuscript.
